# Superficial Bladder Cancer Therapy

**DOI:** 10.1100/tsw.2004.81

**Published:** 2004-06-28

**Authors:** Emmanuel Schenkman, Donald L. Lamm

**Affiliations:** West Virginaia University, Morgantown, WV, USA

## Abstract

Bladder cancer treatment remains a challenge despite significant improvements in preventing disease progression and improving survival. Intravesical therapy has been used in the management of superficial transitional cell carcinoma (TCC) of the urinary bladder (i.e. Ta, T1, and carcinoma in situ) with specific objectives which include treating existing or residual tumor, preventing recurrence of tumor, preventing disease progression, and prolonging survival. The initial clinical stage and grade remain the main determinant factors in survival regardless of the treatment. Prostatic urethral mucosal involvement with bladder cancer can be effectively treated with Bacillus Calmette-Guerin (BCG) intravesical immunotherapy. Intravesical chemotherapy reduces short-term tumor recurrence by about 20%, and long-term recurrence by about 7%, but has not reduced progression or mortality. Presently, BCG immunotherapy remains the most effective treatment and prophylaxis for TCC (Ta, T1, CIS) and reduces tumor recurrence, disease progression, and mortality. Interferons, Keyhole-limpet hemocyanin (KLH), bropirimine and Photofrin-Photodynamic Therapy (PDT) are under investigation in the management of TCC and early results are encouraging. This review highlights and summarizes the recent advances in therapy for superficial TCC.

### INTRODUCTION

Bladder cancer is the fourth most common cancer among men and the eighth most common cancer among women. It is estimated that 54,500 new cases of bladder cancer will be diagnosed in 1997 and 11,700 bladder cancer deaths will occur[1]. The incidence of bladder cancer has increased 36% in the United States from 1956 to 1990 and mortality rates declined 8% between 1980-1995[2]. Intravesical immunotherapy has had a positive impact on disease progression and has influenced the decline in mortality. A proper understanding of the natural history of bladder cancer and significant prognostic factors is critical for management of this disease. Seventy-four percent of cases are superficial at the time of diagnosis, of which 70% are stage Ta and 30% are stage T1[3]. Low-grade non-invasive tumors may be treated with resection and fulguration. However, despite complete tumor resection, two thirds of patients will develop tumor recurrence in five years and by 15 years 88% of patients will develop a recurrence[4]. Progression from superficial bladder cancer to deep muscle invasion occurs in 15% of patients[5,6]. The high rate of tumor recurrence and potential progression provides an opportunity to institute chemoprevention or prophylactic therapy. In this review we highlight the recent advances in intravesical therapy of bladder and summarize the important role of intravesical immunotherapy in the management and prophylaxis of superficial transitional cell carcinoma (TCC) of the urinary bladder.

### INDICATIONS FOR INTRAVESICAL THERAPY

Since its introduction in the late 1950ís, intravesical therapy has been used in the management of superficial bladder cancer with three specific goals. These include eradicating existing/residual tumor, preventing recurrence of tumor after complete bladder tumor resection, and preventing progression of disease. The objectives of intravesical therapy should be tailored to the patient. Intravesical therapy is most effective when tumor burden is minimized by transurethral resection of papillary disease and/or fulguration of visible areas of carcinoma in situ (CIS). The suspected biologic behavior of the patient's tumor remains an important determinant factor in the decision of intravesical therapy. Consequently, a grade III tumor at high risk for recurrence and progression constitutes an accepted indication for intravesical therapy. In the absence of other risk factors for progression, intravesical therapy is not required for grade I/Ta (0) lesions which have a progression rate of only 2-4%[7]. However, multifocal Ta disease with or without CIS is a relative indication for intravesical therapy[8]. Stage T1 disease, irrespective of grade, has demonstrated the biological ability to invade, and has a reported progression rate of 29%[9]. Intravesical therapy is therefore justified to prevent progression to muscle invasion. In his analysis of prognostic factors in a cohort of 505 patients with TCC, Lipponen[10] reported that the initial clinical stage and grade remain the main determinant of survival, irrespective of the treatment. CIS has a high risk of disease progression, with an average 54% developing invasive disease at 5 years[11]. Therefore, the presence of even small foci of CIS should be considered as a definite indication for intravesical therapy. Intravesical immunotherapy is now the first line of treatment for diffuse CIS and has replaced cystectomy as the initial therapy. Multifocal superficial disease irrespective of grade or stage is also associated with increased risk of tumor recurrence and progression, and constitutes an indication for adjuvant intravesical therapy. Other relative indications for intravesical therapy include low grade Ta disease recurring within 2 years, persistent positive urine cytology localized to bladder and urothelial dysplasia or severe atypia.

Prostatic urethral involvement with CIS carries a high risk of progression and poor prognosis and should be treated aggressively. The use of intravesical Bacillus Calmette-Guerin (BCG) immunotherapy has effectively spared cystectomy in many of these patients[12]. Intravesical chemotherapy appears to be ineffective in the treatment of the prostatic urethra. Transurethral resection of the prostate is recommended for tumor staging and to open the bladder neck in order to allow BCG to bathe the prostatic urethra. Reports of response to topical chemotherapy in muscle-invasive disease are lacking, therefore such patients are not treated with intravesical therapy.

### INTRAVESICAL CHEMOTHERAPY IN SUPERFICIAL BLADDER CANCER

Intravesical chemotherapy became popular in the 1960ís when thiotepa was shown to reduce tumor recurrence and eliminate one third of papillary tumors[4]. Unlike systemic chemotherapy, responses to topical chemotherapy are proportional to drug concentration rather than drug dose[13]. Responses are also dependent on the duration of exposure which is short and limited by bladder capacity. Cytotoxic drugs are active against DNA in rapidly dividing cells.

In a review of over 4,000 patients enrolled in controlled intravesical trials, Traynelis and Lamm[14] reported that the average net benefit of intravesical chemotherapy over transurethral resection alone is a modest 14% at 1 to 3 years. Of the 23 reported clinical trials, 13 demonstrated statistically significant reduction in tumor recurrence. Most studies show an advantage of chemotherapy in reduction of tumor recurrence for the first two or three years. Melekos[15] reported that epirubicin prevented tumor recurrence in 60% of treated patients versus 41% of controls during a mean follow-up of 32 months, which is relatively a short-term follow-up. However, long term results with thiotepa, doxorubicin and mitomycin C (MMC) demonstrated that the percent of patients suffering recurrence at 5 or more years is just as high, if not higher, in patients receiving intravesical chemotherapy than in controls. Maintenance chemotherapy has been shown to offer no advantage and perhaps even a disadvantage. Oosterlinck[16] reported recently a reduction in tumor recurrence in patients with solitary Ta or T1 tumors treated with a single early postoperative instillation of epirubicin. In 399 patients, tumor recurrence was reduced from 41% in controls to 29% with epirubicin (p=0.015). Intravesical chemotherapy in the absence of tumor cells should not be beneficial since it acts directly on such cells. Therefore, the concept of preventing future urothelial tumor recurrence with intravesical cytotoxic chemotherapy is illogical.

Although intravesical chemotherapy has demonstrated reduction in short-term tumor recurrence rates, it has not altered disease progression. Progression data are available on more than 2,000 patients enrolled in prospective controlled chemotherapy studies[14]. No statistically significant reduction is found in the risk of disease progression with the use of thiotepa, doxorubicin, mitomycin, or epirubicin in those studies. Moreover, the mean rate of progression for those treated with intravesical chemotherapy was 7.5% compared with 6.9% for the control groups[14]. Similar results have been reported by the EORTC and MRC meta-analysis of over 2500 patients. These investigators demonstrated that with a mean follow-up of 7 years, chemotherapy reduced long-term recurrence by 7%, but had no effect on progression[17].

Intravesical chemotherapy can be beneficial in the management of CIS with reported complete response rates range from 34 to 42%. Sekine et al.[18] have reported the results of mitomycin and doxorubicin sequential therapy in 43 CIS patients. Thirty-two patients (74%) achieved complete response (CR), but despite maintenance therapy, with either MMC or doxorubicin, recurrences occurred in 13 out of 32 (41%). Twenty-six of 32 (81%) complete responders remained disease free during a mean follow up 45 (range 10-84) months. Maintenance therapy failed to show any positive impact on recurrence rate. Three of the 32 complete responders and 5 of 11 nonresponders suffered progression including invasive cancers in four, metastatic disease in two and both conditions in two.

The reasons for the inability of intravesical chemotherapy to affect progression or enhance long-term reduction in recurrence are under investigation. Wientjes et al.[19] reported that the putative variable and inconsistent response of intravesical MMC might be due to physicochemical and hemodynamic factors such as incomplete bladder emptying at treatment, low urine pH, or dilution by constant production of urine. Results of their ongoing Phase III clinical trial will indicate whether or not controlling these variable improve cytotoxic efficacy of mitomycin .

### MECHANISM OF ACTION

BCG is currently the most effective intravesical agent for the treatment and prophylaxis of superficial bladder cancer. BCG is recognized as a nonspecific immune stimulant. Intravesical BCG induces inflammation of the bladder with infiltration of a broad range of cell types. BCG may activate macrophages, T lymphocytes, B lymphocytes, natural killer cells (NK), and killer cells[20]. Intravesical BCG immunotherapy results in cytokine production, including interleukins 1(IL-1), 2(IL-2), and 6(IL-6), interferon gamma, and tumor necrosis factor alpha (TNF-a)[21], which can be measured in the urine for many hours after instillation. McAveray et al.[22] reported that BCG induces a local Type II immunologic response which may be mediated by Interleukin (IL) 4; IL-4, IL-10, the later cytokines may suppress cell-mediated responses. These cytokines also cause a shift to Type I response with the subsequent development of a protective antitumor response. Ratliff et al.[23] investigated the role of $CD_4$ and $CD_8$ lymphokines in the antitumor response of BCG and reported that there is no evidence of induction of protective systemic immunity after BCG. However, they reported a requirement of T-lymphocytes, and $CD_4$ and $CD_8$ subsets in BCG-mediated antitumor activity. They concluded that BCG-mediated antitumor activity is a localized phenomenon. BCG stimulates cytokine production, and this in turn enhances NK cell activity, which increases after BCG immunotherapy[24,25]. Conti et al.[26] reported that immunotherapeutic effects of BCG in bladder cancer patients are related to its capacity to prime macrophages that enhance the release of TNF-a and IL-I alpha, which are involved in tumor killing. BCG produces a T-cell mediated immune response that has been linked to antitumor activity in both humans and mice[27]. The antineoplastic effect of BCG is most likely the result of a combination of enhanced activity of various arms of the immune system.

After intravesical instillation, live mycobacteria attach to the urothelial lining, facilitated by fibronectin, a component of the extracellular matrix[28]. Integrin is required for the direct attachment and internalization of BCG by bladder tumor cells[28-30]. This process leaves bacterial cell surface glycoproteins attached to epithelial cell membranes, and this antigen is thought to mediate the immune response[31]. Tumor cell motility is also thought to be inhibited by BCG through a mechanism involving the BCG-fibronectin-tumor cell interaction[32]. Bladder biopsies following BCG administration show increased expression of human leukocyte antigen (HLA)-Dr antigen on tumor cells and infiltration of tumor and stroma with lymphocytes, predominantly T helper cells, and macrophages. The helper/suppressor ratio in infiltrating lymphocytes is increased. Changes in peripheral blood are also seen, including heightened immunoproliferative response to BCG antigen and production of specific antibody[33-34].

### PRINCIPLES OF BCG IMMUNOTHERAPY

To use immunotherapy effectively in the management of bladder cancer or other malignancy, it is important to consider basic principles and understand the differences between immunotherapy and chemotherapy. Currently chemotherapy is limited in specificity, and basically inhibits or destroys rapidly dividing cells. Generally, tumor cell destruction is proportional to drug concentration so treatments are pushed to the limit of tolerance. In contrast, immunotherapy may be either nonspecific or specific. More often than not, optimal responses to immunotherapy are seen at less than the maximum tolerated dose because high doses invoke complex immune regulatory mechanisms. The typical dose response curve with biological response modifiers such as BCG is therefore bell shaped with optimal response occurring at intermediate doses[35].

The optimal dose of BCG remains to be defined, and may, like the optimal treatment schedule, vary from patient to patient. Current data suggest that intravesical doses between one hundred million (1 x 108) and one billion (1 x 1010) colony-forming units (CFU) are effective, but responses have been reported with doses as low as 10 million CFU or 1 mg BCG[36]. The wide variation in effective clinical doses probably relates to the mode of administration. In intravesical instillation, only those organisms that attach to the bladder wall stimulate an immune response. Therefore, consideration must also be given to avoid administration of medications that can limit the effectiveness of the dose given. Agents that inhibit clot formation reduce fibronectin expression, which may reduce BCG attachment, immune stimulation, and antitumor activity[37-39]. Similarly, concern has been raised that administration of antitubercular antibiotics such as isoniazid (INH), which inhibit intravesical BCG attachment and immune stimulation in the guinea pig model[40], may also reduce the efficacy of BCG therapy. However, Stassar et al.[41] reported that INH does not impair the local immunological stimulation after intravesical BCG. Until additional data becomes available, INH, trimethoprim/sulfamethoxazole, and quinolones should be used with caution in patients receiving BCG. However, these antibiotics should be used without hesitation to treat the side effects of BCG or intercurrent infection.

### EFFICACY OF BCG IMMUNOTHERAPY

Long-term follow-up studies have consistently demonstrated prolonged protection from tumor recurrence by BCG[42-44] as well as increasing evidence to suggest that optimal BCG intravesical immunotherapy also reduces tumor progression and mortality[42-45]. All six clinical studies comparing surgery alone with intravesical BCG immunotherapy demonstrated a highly significant advantage of BCG treatment[45-50] (Table 1). Direct randomized comparisons of BCG immunotherapy with intravesical chemotherapy have

**TABLE 1**
**Effect of Intravesical Bacillus Calmette-Guerin on Recurrence in Control Studies**

| | | CONTROL (TURBT) | | BACILLUS CALMETTE-GUERIN | | |
|---|---|---|---|---|---|---|
| **Authors and References** | **Total Number of Patients** | **Number** | **Recurred #(%)** | **Number** | **Recurred #(%)** | **P Value** |
| Lamm | 57 | 27 | 14 (52%) | 30 | 6 (20%) | <0.001 |
| Herr et al | 86 | 43 | 41 (95%) | 43 | 18 (42%) | <0.001 |
| Herr et al | 49 | 26 | 26 (100%) | 23 | 8 (35%) | <0.001 |
| Pagano et al | 133 | 63 | 52 (83%) | 70 | 18 (26%) | <0.001 |
| Melekos et al | 94 | 32 | 19 (59%) | 62 | 20 (32%) | <0.02 |
| Krege et al | 224 | 122 | 56 (46%) | 102 | 26 (26%) | 0.003 |
| TOTAL | 643 | 313 | 208 (67%) | 330 | 96 (24%) | |

KEY: **TURBT** = transurethral resection of bladder tumor also demonstrated a statistically significant decrease in tumor recurrence rate with BCG compared with thiotepa, doxorubicin, and mitomycin C (MMC)[12, 51-58] (Table 2). The Southwest Oncology Group recently compared TICE BCG (50mg) and MMC (20mg) in 469 randomized high-risk patients with stage Ta or T1 disease[57]. Both treatments were given weekly for six weeks then monthly for one year. A 20mg dose of MMC was previously reported to be the optimum dose[58]. In the MMC arm, tumor reoccurred in 33% of patients with a median time to recurrence of 18.4 months. With a median follow-up of 30 months, 60% of patients in the BCG arm were without tumor recurrence as opposed to 46% of patients in the MMC arm (p=0.017). No toxicity was seen in 18% of the BCG arm or in 30% of the MMC group p<0.003). Melekos et al.[59] reported a recent series of 161 patients enrolled in a three-arm study of intravesical prophylaxis with epirubicin versus BCG versus transurethral resection (TUR) alone. The authors reported that 60% of epirubicin-treated patients, 68% of BCG-treated patients, and 41% of control subjects remained free from recurrences at a median follow-up of 33 months. Epirubicin and BCG were both superior to TUR alone; however, BCG was significantly better than epirubicin in preventing recurrence of stage T1 and high-grade tumors. Cookson and Sarosdy[60] also demonstrated the effectiveness of BCG in high-risk stage T1 patients in their trial; 91% of those treated with intravesical BCG immunotherapy were free of disease at a mean follow-up of 59 months.

The effect of BCG on tumor progression has been investigated in three randomized studies, each of which found a statistically significant reduction in progression to muscle invasion or metastasis[43,45,61]. Lamm demonstrated a reduction in progression to muscle invasive disease in 8% of controls compared to 3% in the BCG group[45]. This positive impact on progression has resulted in improved survival. A controlled trial from Memorial Sloan-Kettering showed persistent reduction in both tumor recurrence and progression after ten years follow-up[62]. However, the reduction in tumor progression did not extend to fifteen years. Overall, 53% of high-risk patients had progression with a disease-specific survival of 63%. Thus, even after apparent successful treatment with BCG, patients remain at risk for progression, recurrence, and mortality and require vigilant long-term surveillance. In another report, Herr et al.[61] reported that within a median follow-up of eight months, mortality rate was reduced from 32% in TUR alone patients to 14% in BCG-treated patients (p<0.032). Herr and associates reported that BCG improved a five-year survival to 87% versus 63% for TUR (p=0.016)[44]. This author has also reported that

**TABLE 2**
**Recurrence in Controlled Comparison Trials**

| Study/References | BCG | Thiotepa | Doxorubicin | Mitomycin C | P Level |
|---|---|---|---|---|---|
| Brosman | 0 | 47% | | | <0.01 |
| Rodriques Netto and Lemos | 7% | 43% | | | <0.01 |
| Martinez-Pineiro et al | 17% | 36% | 43% | | <0.01 |
| BCG/Thiotepa Average | 7% | 42% | | | |
| Lamm et al | 63% | | 83% | | <0.02 |
| BCG/Doxorubicin Average | 38% | | 63% | | |
| Debruyne et al | 30% | | | 25% | NS |
| Juahianen et al | 288% | | | 62% | <-0.01 |
| Rubben et al | 35% | | | 35% | NS |
| Witjes et al | 29% (RIVM) | | | 26% | NS |
| | 34% (TICE) | | | | NS |
| Lamm et al | 20% | | | 33% | <0.01 |
| BCG/Mitomycin Average | 29% | | | 36% | |
| BCG Average | 25% | | | | |
| Chemotherapy Average | 43% | | | | |

*KEY:* **BCG** = bacillus Calmette-Guerin cancer deaths were reduced from 37% to 12% ($p<0.01$) and that the cystectomy rate was reduced from 42% to 26% ($p<0.0001$) in patients with BCG[61]. Nadler et al.[43] demonstrated the durability of a single course of BCG which kept 28% (29/104) of the patients tumor-free at 11 years. However, of the 66 patients who received a second six-week course of BCG for recurrent tumors after failing the intravesical six-week course, 27(41%) remained tumor-free at 11 years. Witjes et al.[62] confirmed that effectiveness of BCG in reducing tumor progression in high-risk patients who had failed prior intravesical chemotherapy for recurrent superficial TCC.

BCG is also effective in the intravesical treatment of CIS. With over 1,000 patients from several series, the average complete response rate of CIS to BCG is in excess of 70%[11]. By comparison, complete response rates for chemotherapy average less than 50%, and in general, fewer than 20% of patients treated with chemotherapy remain disease-free long-term[42].

In contrast to intravesical chemotherapy, data suggest that maintenance therapy with BCG improves long-term results. In a recent report by the Southwest Oncology Group[57] with optimal BCG immunotherapy for recurrent superficial transitional cell carcinoma (CIS, Ta, T1), the complete response (CR) rate was 87% and long-term disease-free status was maintained in 83% of patients. In CIS patients treated with BCG, the complete response at six months post-therapy is increased from 73% to 87% ($p<.04$) with three additional instillations given at six monthly intervals for maintenance[42]. Maintenance BCG using three weekly instillations increased long-term disease-free status from the expected 65% to 83%. In patients with papillary TCC, maintenance BCG given in a series of three weekly treatments at three months, six months, and every six months for three years, dramatically reduced tumor recurrence ($p<.0001$) when compared with a single six-week course[63]. Long-term disease-free status was increased from 50% in the induction-only group to 83% in the maintenance therapy group ($p<0.000001$). More importantly, this maintenance therapy has resulted in statistically significant improvement of patient survival as compared to induction-only. In 391 randomized patients, the excellent 86% survival at four years observed with induction therapy was improved to 92% in patients receiving maintenance BCG ($p<0.04$)[63]. The current recommended maintenance BCG regimen ñ and the only regimen found to be superior to a single, six-week induction ñ employs three weekly instillations of 105 to 108 CFU of Connaught BCG, three months after initiation of treatment[63]. Three weekly instillations are repeated at six monthly intervals for three years. The second or third weekly maintenance treatment is given only if the preceding instillation was without increased side effects. Investigators have reported that in low-dose BCG, 27mg/3x108 CFUs were efficacious, yielding a CR of 84%[48], and some have seen a reduction in toxicity.

It has been suggested that the efficacy of BCG can be improved further by high-dose vitamins[67]. Lamm et al.[65] reported that daily high-dose vitamins A, B6, C, and E (Oncovite, Mission Pharmacal, San Antonio, TX) versus recommended daily allowances (RDA) produced further protection from recurrence in patients treated with BCG. The five-year estimates of tumor recurrence were 91% in the RDA group and 41% in the megadose vitamin group. Overall recurrence was 24 of 30 (80%) patients in the RDA group, and 14 of 35 (40%) in the high-dose arm[64]. Further research is needed to confirm this study and identify which specific vitamins offer the protection from tumor recurrence. Other attempts to improve BCG immunotherapy, such as the addition of intradermal BCG inoculation, have not yet been successful[64].

### COMPLICATIONS OF BCG INTRAVESICAL THERAPY

Intravesical BCG presumably stimulates an immune response to the tumor and thus is associated with unique side effects. Dysuria and urinary frequency are expected as a consequence of the inflammatory response, and cystitis is the most frequent adverse reaction-occurring in up to 90% of cases[65,66]. Hematuria may occur with cystitis and is seen in one-third of patients[66]. Irritative bladder symptoms are unlikely in the week after the first intravesical BCG[66]. Side effects of BCG generally increase with successive treatments, unless the dose of antibiotics is reduced or prophylactic antibiotics are given. Patients with symptoms lasting more than 48 hours can be treated with 300mg INH daily[69]. This treatment is continued only while the symptoms of hematuria and cystitis persist and is reinstituted one day before subsequent BCG instillation and continued for three days. According to Stassar and associates[41], INH does not impair the local immunological stimulation after intravesical BCG or the efficacy of BCG. BCG treatments are postponed until all side effects from previous instillations have resolved. BCG is a live organism, and even though virulence has been dramatically attenuated, regional or systemic infection may occur. BCG organisms usually are gone within a few days of instillation but have been reported to persist in the urinary tract for at least 16.5 months after intravesical BCG[68]. Initial estimates of the incidence of BCG sepsis were in the range of 0.04% and 10 patients died following intravesical BCG[65]. The incidence of sepsis has dropped dramatically after the precaution of not administering BCG after traumatic catheterization or in the presence of continued symptoms of BCG infection. When BCG sepsis does occur, we now recommend INH 300mg, rifampin 600mg, and prednisone 40mg daily. Prednisone is continued until sepsis abates and is then tapered gradually over the next two to four weeks. Rifampin and INH are continued for three to six months, depending on the severity and duration of the reaction. Animal studies[67] have confirmed that this regimen significantly improves survival and no patient receiving this regimen has died of BCG sepsis. The diagnosis of BCG sepsis is made by clinical presentation with high fever, shaking chills, and then hypotension. It is important to proceed with antibiotic treatment without waiting for culture results when systemic BCG infection is suspected. Typically, cultures are negative, even in the face of clinical sepsis. Molecular techniques to identify BCG DNA may prove useful in the future[69].

## OTHER IMMUNOTHERAPEUTIC AGENTS:

### Interferons

Interferons (IFNs) are host-produced glycoproteins that act to mediate immune responses through antiviral, anti-proliferative, and immunoregulatory activities. Torti et al.[70] reported 25% CR in patients with recurrent papillary TCC, and 32% CR and 26% partial response (PR) ñ persistent positive cytology ñ in 19 patients with refractory CIS treated with intravesical IFN-alpha. In a multicenter randomized study, Glashan[71] found tumor response to INFa was dose dependent: there was a 5% CR observed with a dose of 10 x 106 units, and a 43% CR with a dose of 100 x 106 units in patients treated for CIS. In our recent review, IFN-alpha prophylaxis in TCC resulted in a freedom from recurrence in 21-62% of patients within median follow-ups of 6-36 months (Riggs D, Nseyo UO, Lamm DL, unpublished observations). Adverse reactions following intravesical IFN-alpha therapy are relatively mild and include flu-like symptoms of fever, chills, fatigue, and myalgia, which occur in up to 27% of patients.

### Keyhole Limpet Hemocyanin

Keyhole-limpet hemocyanin (KLH), a highly antigenic respiratory pigment of the mollusc Megathura cranulata, is a nonspecific immune stimulator that also has been investigated as an intravesical agent. Jurincic et al.[72] reported that KLH was better than MMC in prevention of superficial TCC recurrence. Flamm et al.[73] compared KLH to ethoglucid for prophylaxis in patients who failed intravesical chemotherapeutics agents, and reported no difference in efficacy. Lamm et al.[74] reported a CR in 25/51 (45%) patients and a PR in 12 (21%) patients with 2mg, 10mg, or 50mg of intravesical KLH for six weeks. The best responders were patients with CIS: of 19 CIS patients, 11 (58%) had a CR. Ten (50%) of 20 patients with papillary TCC demonstrated a response, and 4 (33%) of the 12 patients with both forms of bladder cancer showed response. Wishahi et al.[75] reported that KLH reduced tumor recurrence by 60% in bilharzial bladder with papillary TCC. The advantage of KLH is its apparent lack of toxicity. Lamm et al.[76] reported that crude preparation of KLH offered greater antitumor activity than the purified KLH compound, although this has not been investigated in clinical trial.

### Bropirimine

Bropirimine, an oral immunomodulator, has shown efficacy in therapy of bladder and upper tract TCC. Bropirimine is an aryl pyrimidine with broad spectrum immunostimulatory activity. The spectrum of activity includes induction of endogenous interferons, IL-I, and TNF. Bropirimine also stimulates B-cell proliferation, NK cells, lymphokine-activated killer cells (LAK), and macrophage activity. Sarosdy et al.[77] reported that bropirimine induced a complete response in 27 of 52 (52%) patients treated for residual disease. The best responders were patients without prior intravesical therapy. Of those 10 patients, seven (70%) had a complete response. The median follow-up for the series was 12 months and toxicity was dose related. In a separate investigation, Sarosdy et al.[78] reported the results of 25 patients with unilateral or bilateral positive cytology with negative retrograde pyelography. Ten of 19 (53%) evaluable patients showed negative cytology following oral bropirimine therapy. Four patients showed a cytologic conversion within three months, and the remaining six patients showed this conversion at six months. The duration of response ranged from 3 to 30 months; the duration of therapy was six months in most cases. Two of the responders relapsed within the follow-up period[78].

### Photofrin-Mediated Photodynamic Therapy (PDT)

Photofrin-Mediated Photodynamic Therapy (PDT) involves intravenous administration of photosensitizers with subsequent in situ intravesical activation by use of whole bladder laser therapy (WB-PDT) with visible light (630nm). Many compounds are being evaluated as potential photosensitizers[79]; however, PHOTOFRIN, Porfimer Sodium (QLT Phototherapeutics, Inc., Vancouver, BC, Canada) is the only photosensitizer which is approved by the U.S. Federal Drug Administration for clinical use. PDT has been evaluated in therapy of recurrent superficial papillary TCC and refractory CIS, in the prophylaxis of recurrent superficial TCC[80]. In studies involving 51 patients with TA and/or T1 TCC, CR occurred in 41%, while another 39% demonstrated PR following a single PDT treatment. For papillary TCC, tumor size was a factor ñ CR was observed only in tumors less than 2 cm in diameter. In a multicenter randomized trial involving 36 patients, preliminary findings on 24 patients indicate a reduction in recurrence from 83-33% (net benefit of 50%) with a single prophylactic PDT treatment following a complete transurethral resection of the bladder tumors[80]. The median time to tumor recurrence increased from 3-13 months with the addition of a single adjuvant PDT treatment. Long-term data on prevention of recurrence and progression are lacking.

An important application of PDT treatment is in the management of refractory/recurrent CIS. In a single whole bladder, PDT treatment produced a complete response in 88% of the patients, and only 25% of the patients experienced recurrence during follow-ups ranging from 3-55 months. Recently Nseyo et al.[81] reported on a multicenter clinical trial involving 35 patients who received a single PDT treatment for refractory CIS as an alternative to cystectomy. All patients had failed standard regimens of at least two intravesical therapies including BCG. The authors showed that PDT treatment induced complete responses in 52% of these highest risk patients, and spared 58% of the patients radical cystectomy.

The mechanisms of action of PDT include direct cytotoxicity mediated by singlet oxygen and superoxide radicals; vascular endothelial damage with thrombosis and hypoxia; and intense local inflammation associated with immune response. Consequently, PDT treatment induces symptoms of cystitis (post-PDT syndrome) urinary frequency, urgency, nocturia, suprapubic pain, and bladder spasm. The intensity of duration of these symptoms depend directly on PDT dose (light and PHOTOFRIN), extent of detrusor damage from previous treatments, intensity of a acute inflammation, and CIS (which enhances PHOTOFRIN retention). The most severe adverse reaction of PDT treatment is permanent bladder contracture which has been reported in 4-24% of patients[80,81]. With proper patient education and selection, the problem of PHOTOFRIN -induced skin photosensitivity has been minimal. However, avoidance of direct exposure to sunlight is required up to six weeks after PHOTOFRIN injection. Introduction of new photosensitizers and simplification of WB-PDT laser light delivery may lead to wide clinical application of PDT in the management of bladder cancer.

### CONCLUSION

Intravesical BCG remains the most effective therapy in the management and prophylaxis of superficial TCC of the urinary bladder. BCG intravesical immunotherapy has improved tumor recurrence rates, disease progression rates, and has prolonged survival of patients with this disease. Intravesical chemotherapy, on the other hand, has reduced tumor recurrence rates but has had no positive impact on disease progression or survival. Although results from newer intravesical therapies such as IFN, KLH, bropirimine, and PDT are encouraging, to date they have only proven to be useful in the therapy and prophylaxis of superficial TCC and long-term data on the prevention of recurrence, disease progression, and survival are unknown.
